# Supplementary material for: Path integration maintains spatial periodicity of grid cell firing in a 1D circular track
Source: Nat Commun. 2019 Feb 19;10:840. doi: 10.1038/s41467-019-08795-w (PMC6381105; doi:10.1038/s41467-019-08795-w)
Supplement: Supplementary file 1 — Supplementary information [file 41467_2019_8795_MOESM1_ESM.pdf]

Supplementary information

Path integration maintains spatial periodicity of grid cell firing in a 1D circular track

Jacob et al.

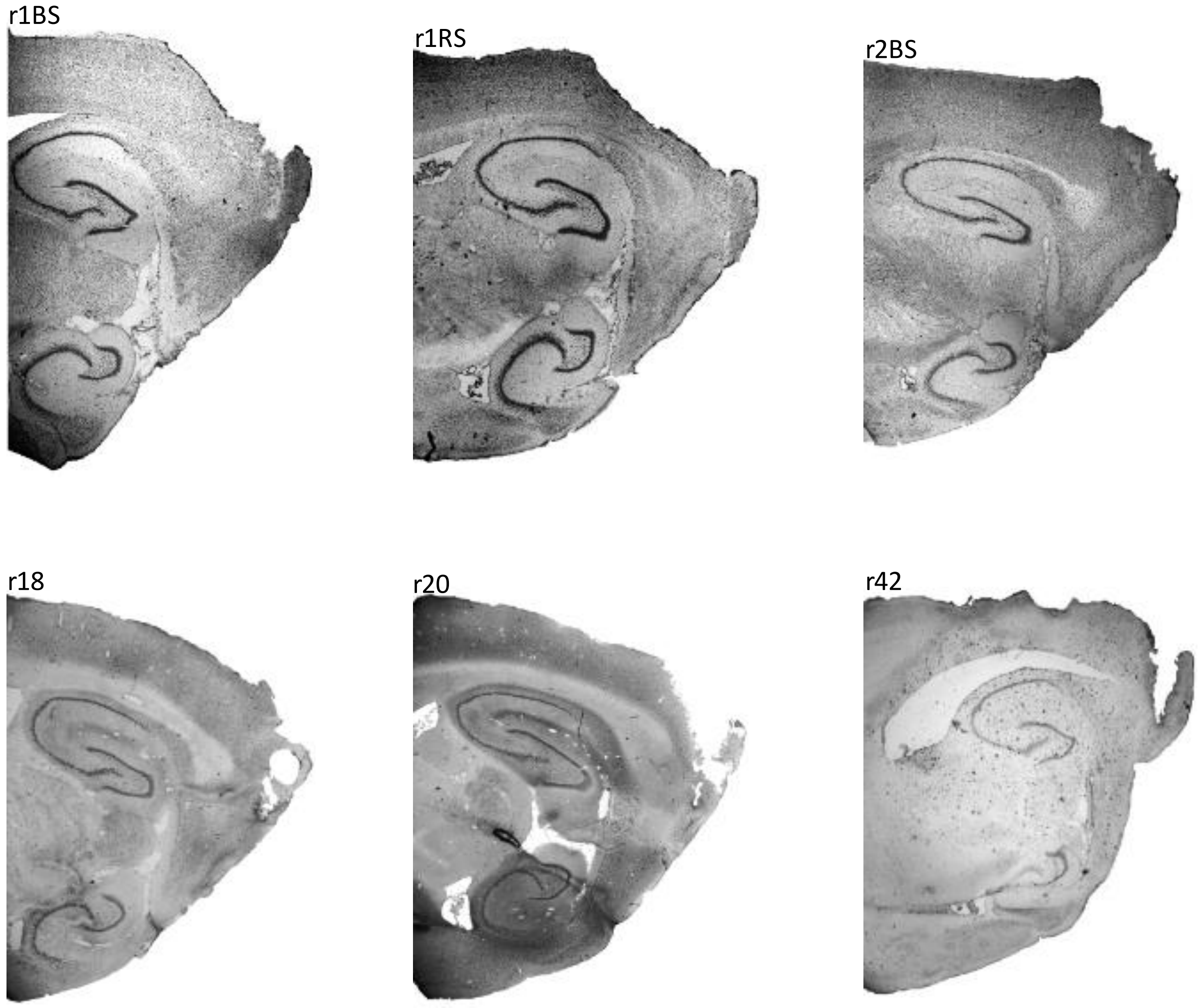

Supplementary figure 1: Histology. Sagittal cresyl violet-stained brain sections showing the recording locations in the MEC of the 6 animals used in this study.

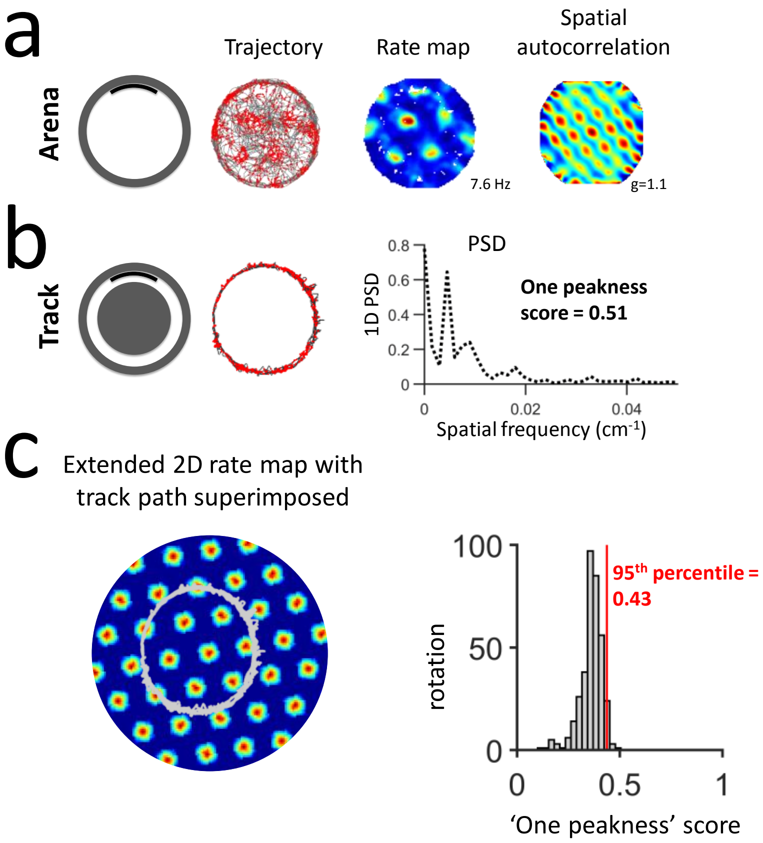

Supplementary figure 2: Power spectral density of grid cell firing activity. a: Example of a grid cell recorded in the arena (from left to right, rat trajectory with spike locations, rate map and spatial autocorrelation; the peak of the firing rate and the grid score 'g' are indicated). b: Firing activity of the same grid cell in the track (from left to right, rat trajectory with spike locations and the power spectral density PSD). The 'one peakness' score was calculated by dividing the area under the higher peak by the total area of the PSD. c: Control procedure. For each cell we superposed the path of the animal in the track to the extended 2D grid map (shown in panel a) and we calculated the 'one peakness' score. The procedure was repeated by shifting the animal path on the extended 2D so as to cover all bins in a triangular lattice. The 95th percentile of 'one peakness' scores distribution was used as statistical threshold.

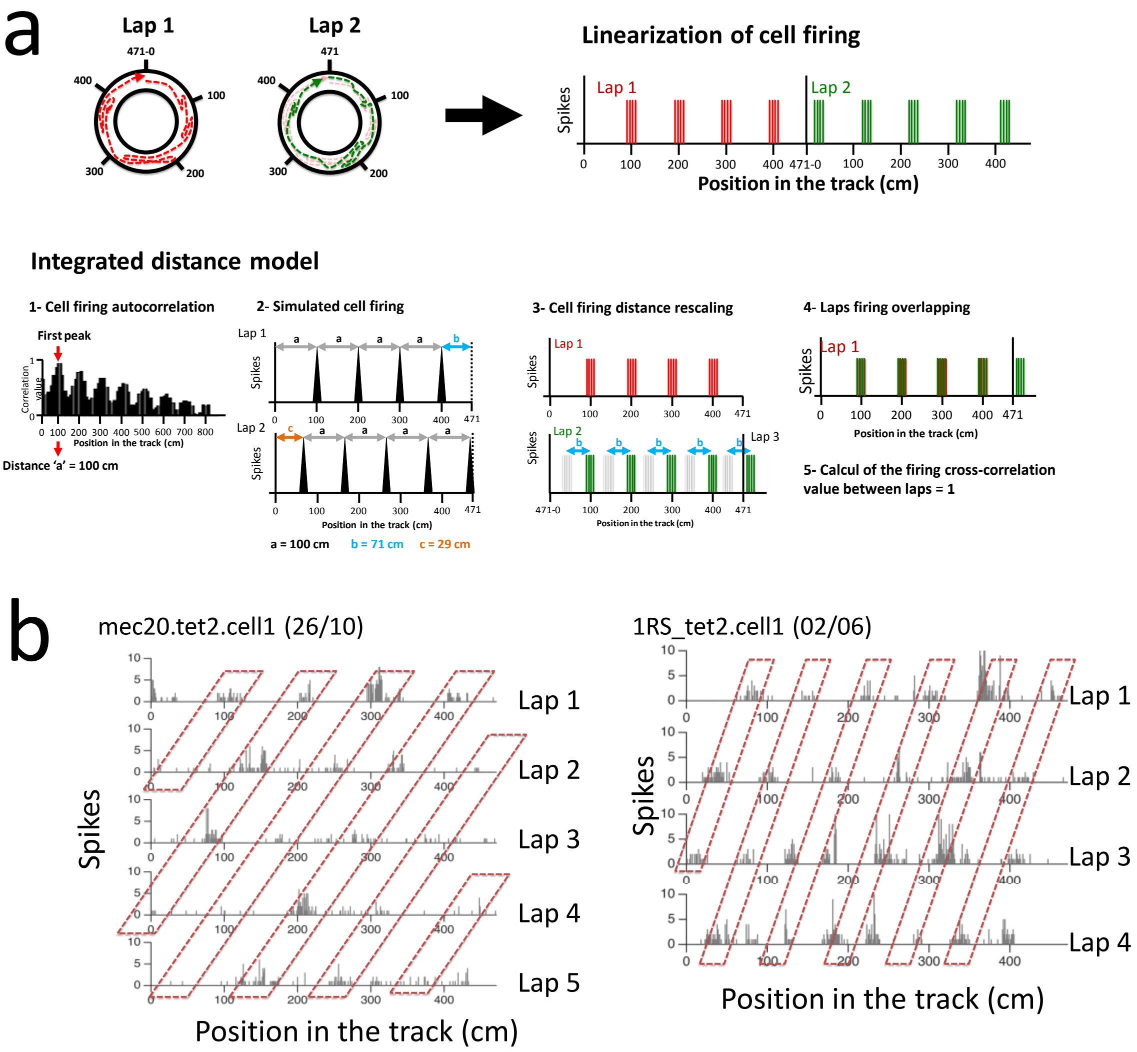

Supplementary figure 3: a: Steps of the procedure used to calculate cell's integrated distance firing correlation between laps in the track. The autocorrelogram of the linearized cell firing was computed, and the distance of the first peak was extracted (1). This distance was used to simulate cell firing lap to lap and to calculate for each lap the residual distance 'b' (2). Residual distances were used to shift real cell firing from lap to lap (3). At the end of this rescaling procedure, the correlation between the firing of pairs of laps was computed. b: Examples of the firing activity of two different grid cells from 2 rats linearized according to the path integrated distance model. Note that the firing fields are shifted lap after lap.

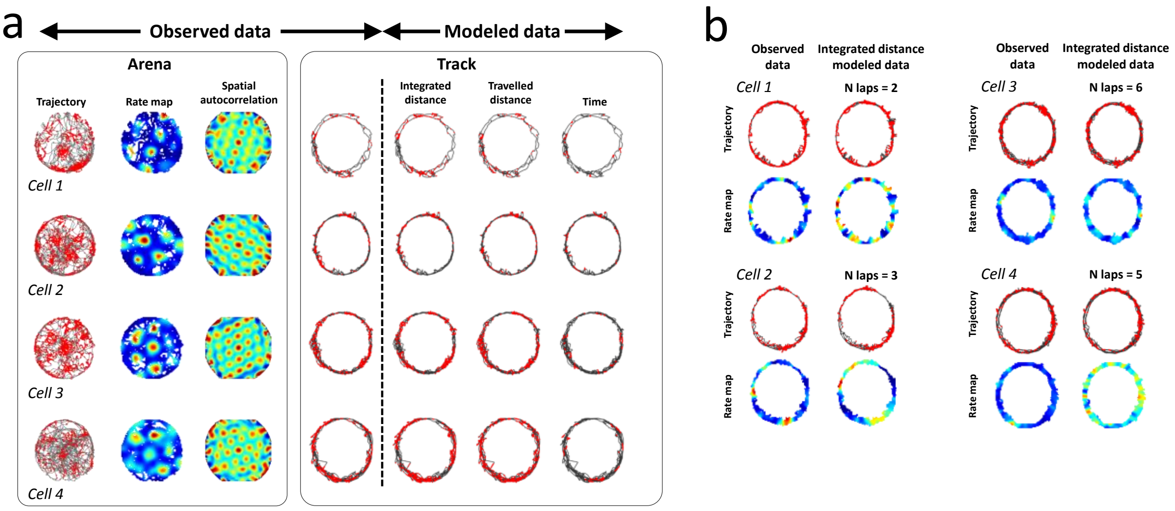

Supplementary figure 4: a: Examples of the rat trajectory with spike locations of four grid cells recorded from 3 different animals in the arena and the track (observed data), and after reconstruction (modeled data) of cell activity according to three different models (i.e. path integrated distance, travelled distance and time). b: Spatial firing fields are clearly identified in the rate map if animals ran a small number of laps (Cell 1 and 2). In contrast, rate maps show more diffuse activity without clear fields when animals performed more laps (Cell 3 and 4).

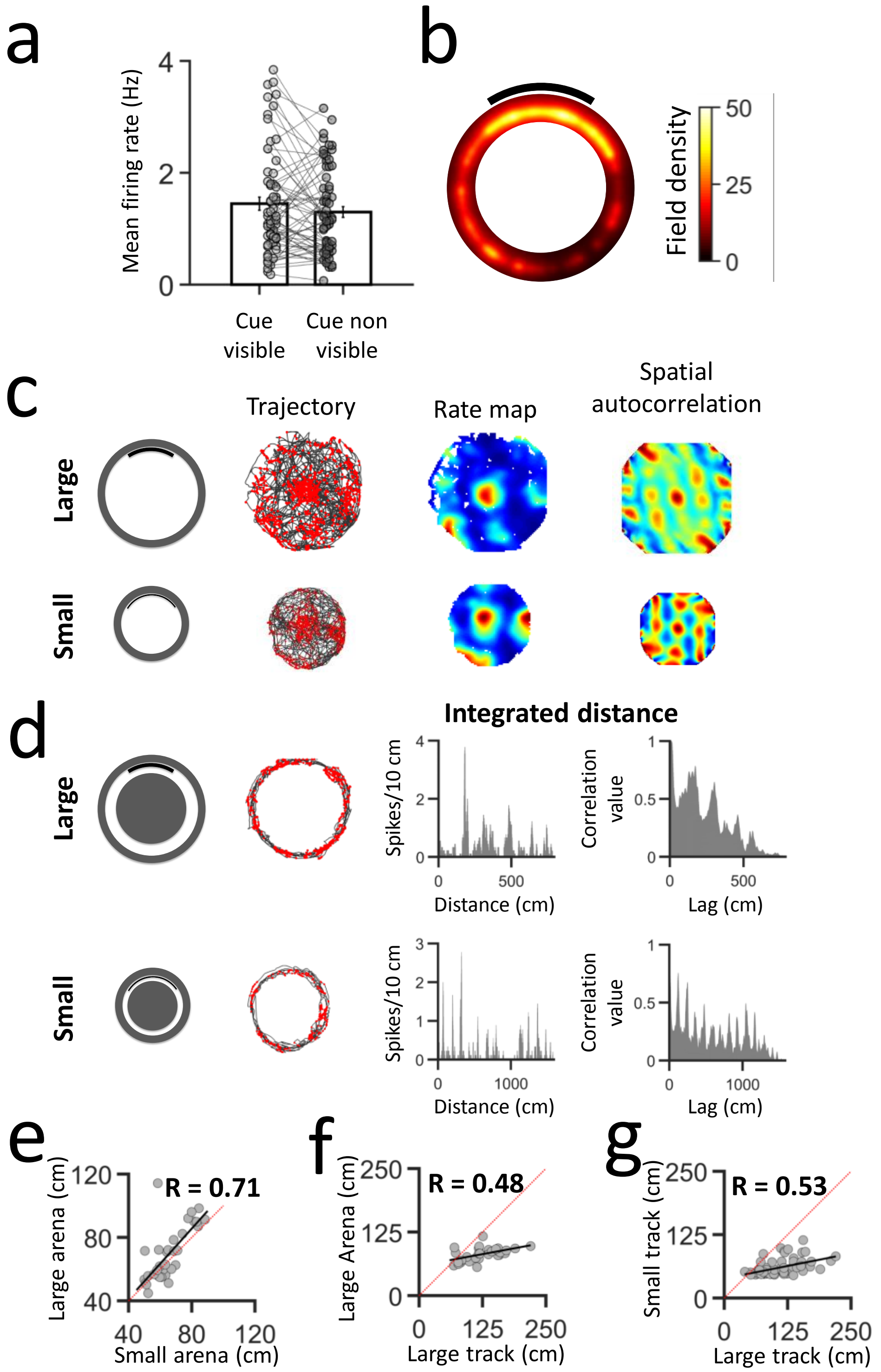

Supplementary figure 5: a: Average mean firing rate from all grid cells in the visible-cue and non-visible cue region of the track. b: Density plot of firing field positions in the track for all recorded grid cells (color-coded from black (no field was detected) to red to yellow to white (50 fields were detected)). A greater number of fields was detected in the visible-cue region

compared to the non-visible cue region of the track. c-d: Example of one grid cell recorded in large and small arenas (c) and tracks (d). First column: rat trajectory with spike locations, rate map and spatial autocorrelogram of the grid cell activity in the arena. Second column: rat trajectory with spike locations and rate map from the same cell recorded in the track. Third column: firing activity linearized according to the path integrated distance model, autocorrelogram and Toeplitz matrix. Note that the number of peaks in the autocorrelogram for the small track is the double of that observed in the large track, indicating that the distance between fields is smaller in the small track compared to the large track. e-f-g: Scatter plots showing the relation between the inter-field distance in the small and large arenas (e), in the large arena and large track (f), and in the small and large tracks (g). Pearson- correlation R values are shown.

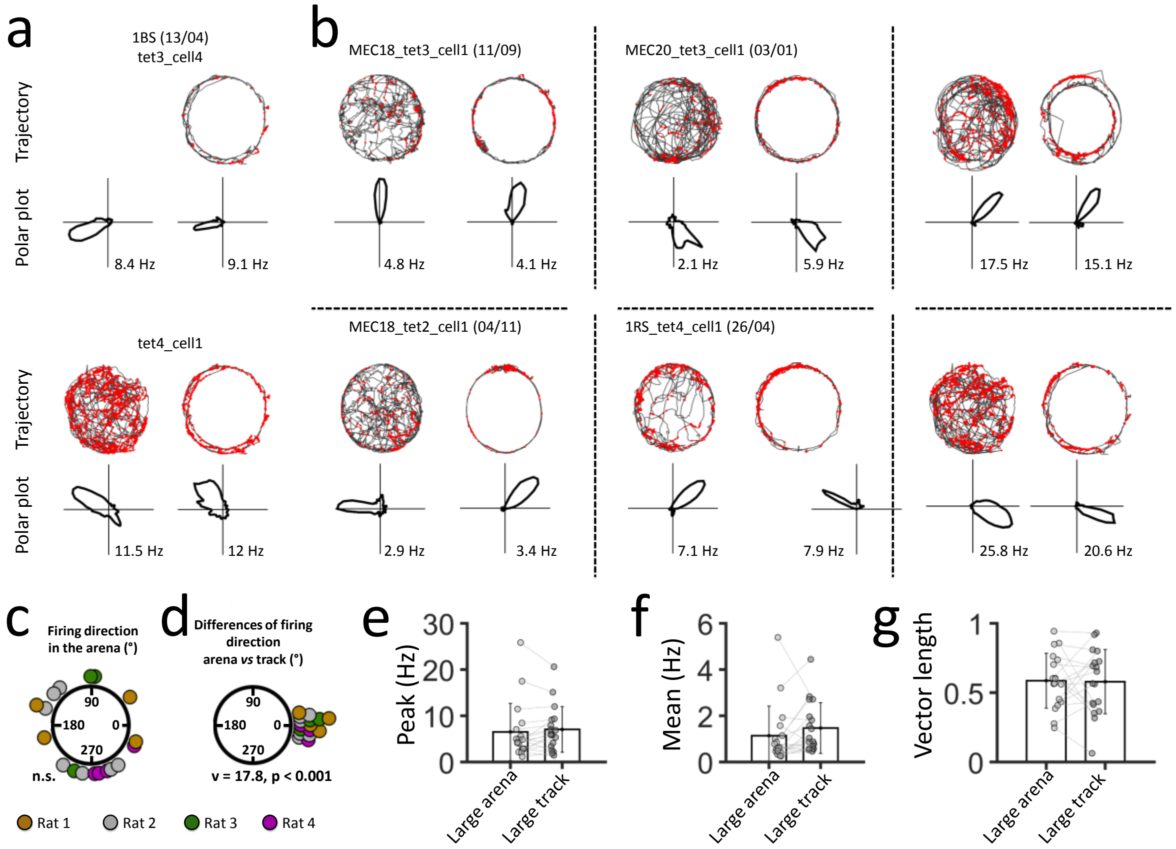

Supplementary figure 6: a -b: Polar plots of two simultaneously recorded head direction cells in the arena and in the track (a) and six head direction cells recorded from three different animals in the arena and the track (b). Bottom: polar plots of firing rate vs head direction (peak firing rate is reported). In all these examples, the preferred firing direction of head direction cells is stable between the arena and the track. c: Distribution of HD firing directions in the arena; ‘n.s.’ Rayleigh test. d: Distribution of the difference between the firing directions of HD cells in the arena and the track. ‘v’ and ‘p’ indicate the V-test value and significance. e-f-g: Rayleigh vector length, peak firing rate and mean firing rate of HD cells in the arena and the track. Histograms show means ±SD. Data from individual HD cells are shown in grey.
